# Multipotent Cancer Stem Cells Derived from Human Malignant Peritoneal Mesothelioma Promote Tumorigenesis

**DOI:** 10.1371/journal.pone.0052825

**Published:** 2012-12-28

**Authors:** Sheelu Varghese, Rebecca Whipple, Stuart S. Martin, H. Richard Alexander

**Affiliations:** 1 Division of General and Surgical Oncology, Department of Surgery, University of Maryland School of Medicine, Baltimore, Maryland, United States of America; 2 Marlene and Stewart Greenebaum Cancer Center, University of Maryland School of Medicine, Baltimore, Maryland, United States of America; City of Hope National Medical Center and Beckman Research Institute, United States of America

## Abstract

During the progression of malignant peritoneal mesothelioma (MPeM), tumor nodules propagate diffusely within the abdomen and tumors are characterized by distinct phenotypic sub-types. Recent studies in solid organ cancers have shown that cancer stem cells (CSCs) play a pivotal role in the initiation and progression of tumors. However, it is not known whether tumorigenic stem cells exist and whether they promote tumor growth in MPeM. In this study, we developed and characterized a CSC model for MPeM using stably expandable tumorigenic stem cells derived from patient tumors. We found morphologically distinct populations of CSCs that divide asymmetrically or symmetrically in MPeM *in vitro* cell culture. The MPeM stem cells (MPeMSCs) express stem cell markers c-MYC, NES and VEGFR2 and in the presence of matrix components cells form colony spheres. MPeMSCs are multipotent, differentiate into neuronal, vascular and adipose progeny upon defined induction and the differentiating cells express lineage-specific markers such as TUBB3, an early neuronal marker; vWF, VEGFA, VEGFC and IL-8, endothelial markers; and PPARγ and FABP4, adipose markers. Xenotransplantation experiments using MPeMSCs demonstrated early tumor growth compared with parental cells. Limiting dilution experiments using MPeMSCs and endothelial lineage-induced cells derived from a single MPeMSC resulted in early tumor growth in the latter group indicating that endothelial differentiation of MPeMSCs is important for MPeM tumor initiation. Our observation that the MPeM tumors contain stem cells with tumorigenic potential has important implications for understanding the cells of origin and tumor progression in MPeM and hence targeting CSCs may be a useful strategy to inhibit malignant progression.

## Introduction

Cancer stem-(like)-cells with self-renewal and tumor initiating potential have been identified in different tumor types [1]–[3] and recent evidence suggests that these cells play a central role in the progression of malignant tumors. The CSC model describes the existence of a small subpopulation of plastic cells with transdifferentiation potential in tumors. However, recent studies suggest that a major proportion of cells within tumors maintain stem cell properties and even more differentiated cells can be transformed into stem-like cells [4], [5]. If this is the case, elimination of CSCs might not be a useful strategy for the abatement of tumor growth. Therefore, it is important to develop models to understand CSC biology and identify new strategies to prevent malignant tumor progression. The mechanisms that regulate self-renewal of both CSCs and normal stem cells are thought to be similar [6]. Presently, identification and isolation of CSCs have largely been dependent on specific cell surface markers [7], [8], although the expression of such markers is dependent on various factors such as the differentiation state of the cells and niche factors. CD133 has been used as a putative stem cell marker in glioblastoma [9] and colon cancer [10], CD34 expressing tumor epithelial cells as CSCs in cutaneous cancer [11], CD44 expressing cells in breast cancer [12], CD26 positive cells are involved in the process of metastases, invasiveness and chemoresistance in colon cancer [13], CD271 positive cells initiate melanoma progression and metastasis [14] and properties of CSCs were identified in CD24 positive pleural mesothelioma cells [15]. However, there has not been any evidence for the existence of stem-like cells that initiate tumor growth in MPeM. Therefore, we investigated the presence of tumorigenic mesothelioma cells with properties of CSCs in stable cell lines derived from MPeM patient tumors.

Malignant peritoneal mesothelioma originates diffusely within the serosal lining of the peritoneum; it is an aggressive cancer with a marked tendency for regional metastases. At diagnosis, this cancer is generally found at a stage of diffuse malignant progression with a large number of variably sized tumor nodules. Generally, tumors have a poorly vascularized thick inner core surrounded by an outer neovascular area. Treatment options such as surgical cytoreduction and systemic chemotherapy can control tumor progression in some patients but patient death is predominantly due to progressive tumor relapse. We recently reported that activation of phosphatidylinositol 3-kinase (PI3K) and the mammalian target of rapamycin signaling in MPeM is associated with shortened patient survival [16]; these pathways are involved in stem cell activation and proliferation [17], [18]. PI3K signaling is also important for the maintenance of cell polarity in cancer cells [19].

Although the derived MPeM cell lines survive indefinitely during passaging, in this study, we used early-passage cells derived from patient tumors to avoid selection of a particular subset of aggressive tumor initiating cells preserved during prolonged passaging. Here we identified that a major proportion of prospectively generated stable MPeM cells in culture generate floating cells with properties of embryonic stem cells such as asymmetric cell division, self-renewal and multipotency. The randomly selected single MPeMSCs showed clonal progression, phenotypic diversity under defined conditions and transdifferentiation potentials. Therefore, the considerable plasticity of MPeMSCs may contribute to the formation of the developmentally diverse cell population found in tumors and aggressiveness of tumors during the pathological progression. Collectively, our study reveals that MPeM harbor CSCs and tumor progression is due to the self-renewal and differentiation of CSCs.

## Results

### Morphologically Distinct Populations of MPeMSCs that Divide Asymmetrically or Symmetrically and Express Stem Cell Markers

**Figure 1:**
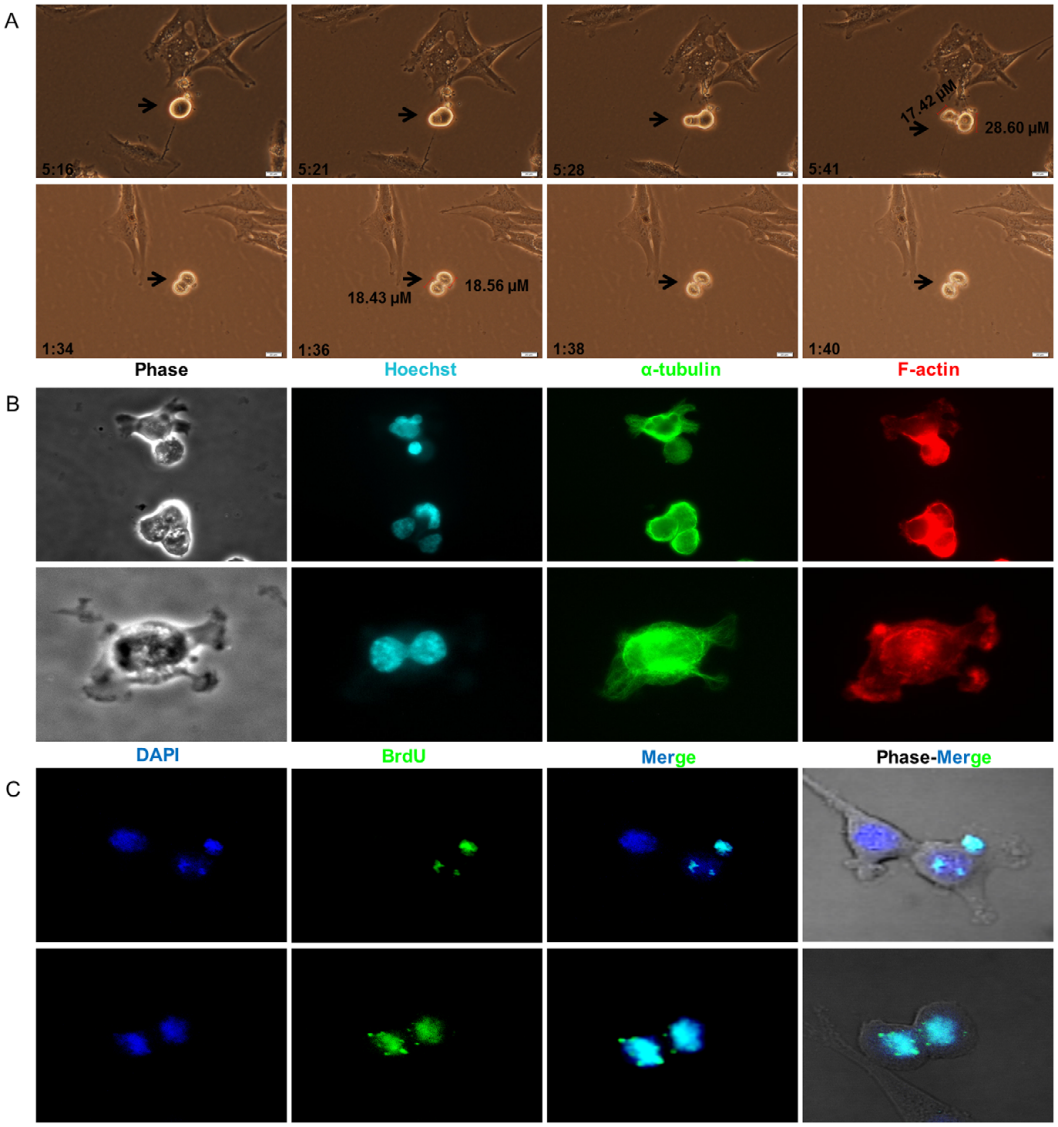
Live and immunofluorescence of malignant peritoneal mesothelioma stem cell (MPeMSC) division. (**A**) Time-lapse images of asymmetric and symmetric cell division in MPeMSCs. Asymmetric cell division (arrows) generates two unequal cells (upper panel); symmetric cell division (arrows) generates two identical cells (lower panel). Cell size shown in µM. Time (hour : minute) noted on the lower left corner. Scale bars: 20 µM. (**B**) Fluorescent images of asymmetric (upper panel) and symmetric (lower panel) cell division in MPeMSCs. Hoechst nuclear staining shows asymmetric and symmetric division of cells. (**C**) DNA is segregated asymmetrically during cell division (upper panel). Cells are grown in BrdU for several passages (the pulse period) and after a long pulse BrdU is removed from the cell culture (the chase); BrdU retention in the dividing cells was studied using anti-BrdU antibody. In an asymmetric cell division, the template DNA labeled with BrdU segregated within the new daughter stem cell whereas the newly synthesized DNA without BrdU label condensed within the differentiating cell. During symmetric cell division BrdU labeled DNA segregated randomly within the daughter cells (lower panel).

**Figure 2:**
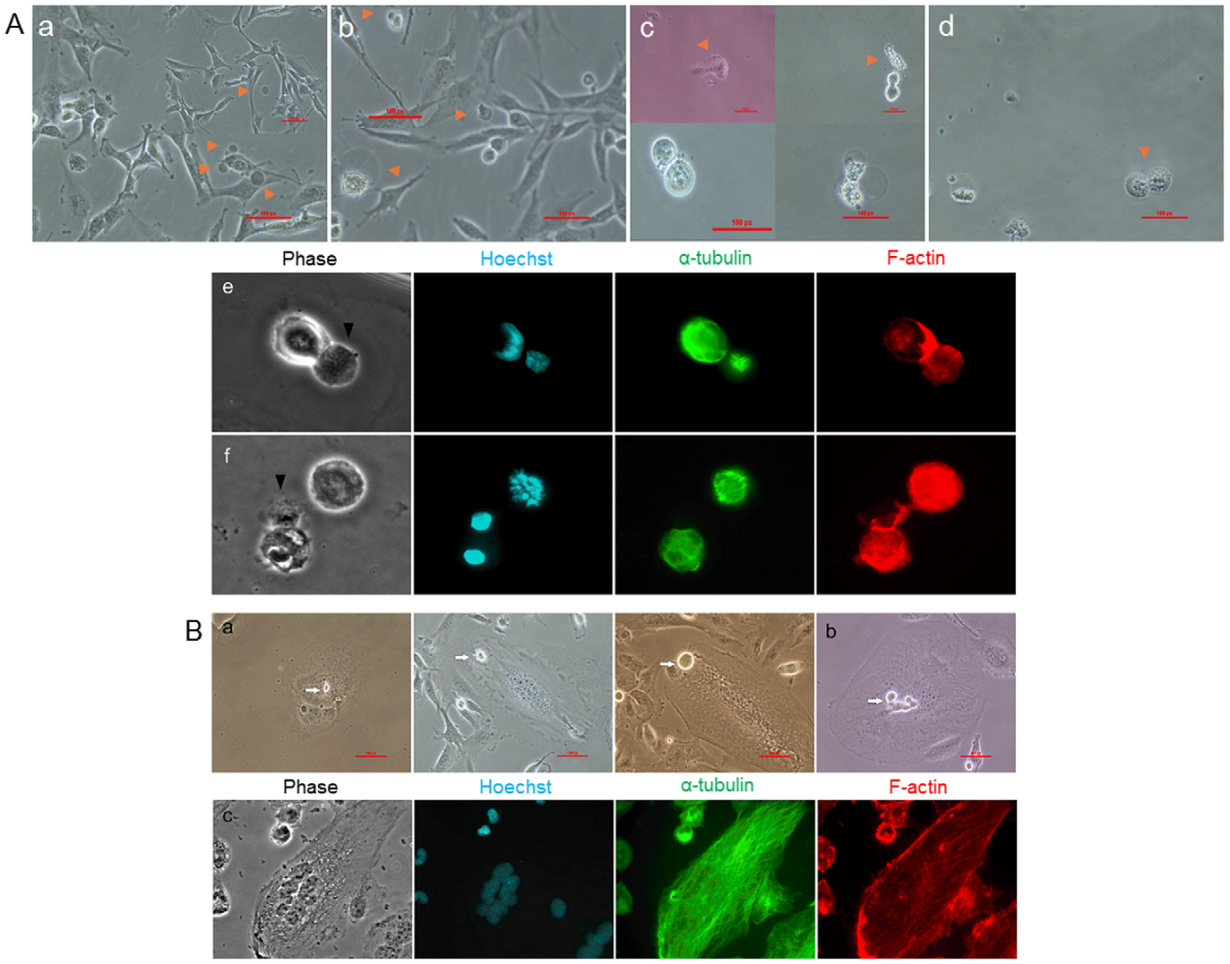
Malignant peritoneal mesothelioma stem cells (MPeMSCs) with distinct morphology. (**A**) Formation of MPeMSCs with capsular covering and possible asymmetric and symmetric stem cell division. **a, b,** CSCs originate from the parental cells as dark bubble-like protrusions (arrows). Cells detach and float in the culture and MPeMSCs ‘hatch-out’ of the capsule (arrows). **c,** Asymmetric cell division in capsular stem cells. Asymmetrically dividing cells originate from the capsule (upper panel, arrows) or cells divide unequally along with the capsule generating two non-identical cells (lower panel). **d,** Symmetric cell division of a capsular stem cell. Scale bars: 100 px. **e, f,** Fluorescent images of asymmetric (e) and symmetric (f) cell division of encapsulated cells. Arrows indicate the outer capsular covering of the cell (**B**) **a,** MPeMSCs originate from the nucleus of a multinucleated cell; the newly formed cell moves through the cytoplasm and is released from the parental cell. **b,** Origin of multiple CSCs from a multinucleated cell. c, Fluorescent images of a multinucleated cell.

Among the stable MPeM cell lines generated, Meso-CL1 was PTEN negative ^(neg)^ whereas Meso-CL2 and Meso-CL3 were PTEN expressing cell lines (**Fig. S1**). Although all three MPeM cell lines generated stem cells with self-renewal that detach and float in culture before adherent growth, Meso-CL1 has the propensity to generate tumor xenografts upon subcutaneous or intraperitoneal implantation in immunocompromised mice and was used to generate an MPeM CSC model. Generally, mammalian CSCs form multiple aggregates of spheroid colonies in culture [20]; colony formation was limited when MPeMSCs were cultured under standard adherent cell culture conditions. However, clonal sphere formation was activated when cells were cultured in a Matrigel plate. Light microscopy studies have identified two morphologically distinct populations of CSCs in MPeM culture; cells with or without an outer encapsulating covering, both exhibited asymmetric or symmetric division. To demonstrate that the isolated cells maintain stem cells properties, we first performed time-lapse studies on live cells in culture and immunofluorescence studies in formaldehyde-fixed cells and found that cells divided both asymmetrically and symmetrically **(** **Fig. 1A, B** **).** In an asymmetric division, cells divided unequally generating a large stem cell and a small cell generally termed as the progenitor cell. Pulse-chase experiments using the thymidine analog BrdU have shown that during asymmetric cell division the template DNA labeled with BrdU, after a long pulse, exclusively segregated within the new stem cell, whereas the newly synthesized DNA condensed within the differentiating cell. In addition, we also found that during symmetric cell division both daughter cells shared the template DNA **(** **Fig. 1C** **)**. Another morphologically distinct population of CSCs was identified in MPeM culture with an encapsulating outer covering that formed from the parental cells as dark bubble-like protrusions. The newly formed cells detached from the parental cell and floated in culture and cells ‘hatched’-out from the outer covering to initiate growth. Because of the floating nature of these cells, time-lapse studies were unsuccessful; however, the light and fluorescent microscopy evidences suggest that the cells also divided asymmetrically or symmetrically **(** **Fig.** **2A** **)**. As found in other cancer cell lines, we have also identified large multi-nucleated adherent cells in cell culture that generate one or more stem cells typically originating from the nucleus without complete cytokinesis of the parental cell **(** **Fig. 2B** **)**.

**Figure 3:**
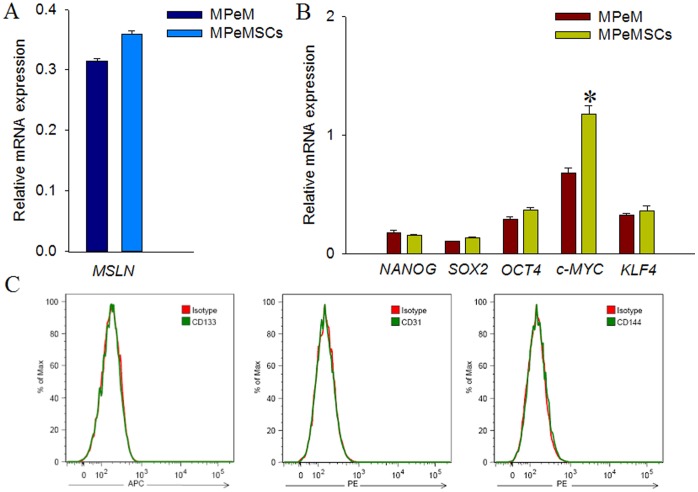
Expression of a mesothelioma marker gene and stem cell markers in malignant peritoneal mesothelioma stem cells (MPeMSCs). (**A**) Relative mRNA expression of mesothelin (MSLN) and (**B**) the stem cell marker genes in parental MPeM cells and in MPeMSCs. c-MYC expression was significantly higher (*p<0.05) in MPeMSCs whereas other stem cell markers showed comparatively similar pattern of expression in both groups. (**C**) Flow cytometry evaluation of human CD133, CD31 and CD144 demonstrated that the proteins are not expressed in MPeMSCs. Bar diagrams are expressed as mean ±SEM.

Next we determined whether the isolated MPeMSCs were truly derived from parental malignant mesothelial cells. We assessed the expression of a known mesothelioma marker, MSLN, and found a robust expression of the gene in both MPeMSCs and parental MPeM cells **(** **Fig. 3A** **)**. The stem cell properties of the isolated MPeMSCs were further determined by the expression of known stem cell markers including NANOG, SOX2, OCT4, c-MYC and KLF4 (**Fig. 3B**). Interestingly, the pattern of expression of early stem cell markers was similar in both parental cells and MPeMSCs whereas c-MYC expression was significantly higher in MPeMSCs compared with parental cells. Conversely, the pluripotent stem cell marker, CD133 and endothelial stem cell markers, CD31 and CD144, were undetectable in MPeMSCs **(** **Fig. 3C** **)**.

### Multiple Lineage Induction in MPeMSCs

**Figure 4:**
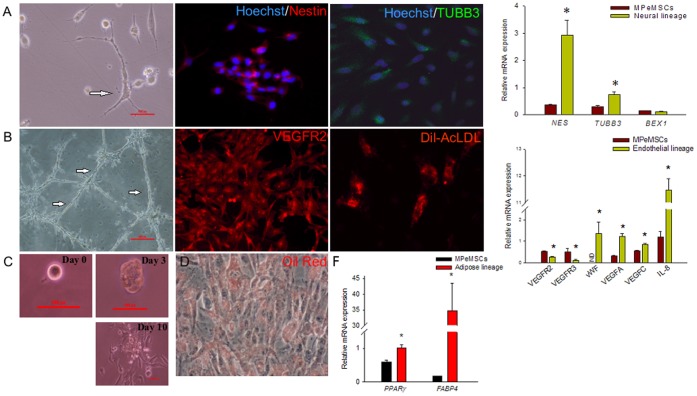
Multilineage differentiation of malignant peritoneal mesothelioma stem cells (MPeMSCs). (**A**) Differentiation of MPeMSCs into neuron-like cells in the neural differentiation medium (arrow). Immunofluorescence and relative mRNA expression studies shows that differentiated neuronal cells express the neural stem cell marker nestin and an early neuronal marker βIII-tubulin (*p<0.01). (**B**) Endothelial differentiation of MPeMSCs on a Matrigel surface form tubular networks (arrows). Fluorescent imaging shows that cells express the endothelial stem cell marker, VEGFR2, and uptake of Dil-AcLDL by differentiated endothelial cells. Relative mRNA expression studies demonstrates that during endothelial differentiation VEGFR2 and VEGFR3 expression decreased significantly (*p<0.05) whereas the expressions of endothelial cell markers, vWF, VEGFA, VEGFC and IL-8 significantly increased (*p<0.001). (**C**) A single MPeMSC form a colony sphere on a Matrigel surface followed by extensive proliferation of MPeMSCs. Scale bars 100 px. (**D**) MPeMSCs differentiated into adipocytes (Oil Red) and (**F**) express adipocyte markers PPARγ and FABP4 (*p<0.01) in adipose differentiated cells compared to controls. Bar diagrams are expressed as mean ±SEM; ND, not detectable.

Multipotency is the hallmark of stem cells. However it has not been conclusively demonstrated that CSCs can attain multiple fates upon lineage induction. To demonstrate the ability of MPeMSCs to achieve multiple fates, CSCs were cultured in the neuronal, vascular and adipose differentiation conditions. When sub-confluent adherent MPeMSCs were treated with trans-retinoic acid, followed by the addition of neural differentiation medium, cells differentiated into neuron-like cells with typical neuronal morphology. Fluorescent imaging and qPCR studies of mRNA expression showed that the neuronal lineage-induced cells expressed neural stem cell marker NES and an early neuronal marker TUBB3; however, BEX1 showed no significant difference between MPeMSCs and neural differentiated cells **(** **Fig. 4A** **)**. The floating neural stem cells derived from the neuronal differentiated cells were collected and cultured for several generations to demonstrate self-renewal and many of these cells displayed typical neuronal morphologies.

We next assessed the ability of MPeMSCs to form endothelial cells. MPeMSCs express VEGFR2, an endothelial progenitor cell marker. Embryonic stem cells positive for VEGFR2 form vascular progenitors and transform into mature blood vessels [21]. To study the differentiation of MPeMSCs into endothelial lineage, cells were grown in culture dishes coated with basement-membrane matrix components (Matrigel). On Matrigel, cells induced endothelial cell differentiation and formed tubular networks. Gene expression studies in endothelial lineage-induced cells compared with MPeMSCs revealed that VEGFR2 and VEGFR3 expression were significantly reduced in cells during endothelial differentiation whereas the expression of vWF, VEGFA and VEGFC were upregulated in differentiating cells. Similarly, the pro-angiogenic factor, IL-8 showed a 10-fold increase during endothelial cell differentiation compared with MPeMSCs. To further validate the endothelial differentiation, cells were incubated with low-density lipoprotein, Dil-AcLDL, and found uptake of lipoproteins by endothelial differentiated cells (**Fig. 4B**). In the Matrigel MPeMSCs also form colony spheres followed by the extensive proliferation of cells (**Fig. 4C**). The proliferating cells generate multiple clonal spheres in *in vitro* cell culture facilitating the rapid proliferation of endothelial progenitor cells.

To determine whether the MPeMSCs differentiate into adipocytes, cells were exposed to trans-retinoic acid followed by adipose differentiation medium and tested for intracellular neutral lipid accumulation. Lipid deposits were identified in adipose differentiated cells. Control and differentiated cells were further analyzed for known adipocyte markers using qPCR and found a significant upregulation of PPARγ and FABP4 (**Fig. 4D, F**).

### Tumor Formation from Single Cell MPeMSCs

**Figure 5:**
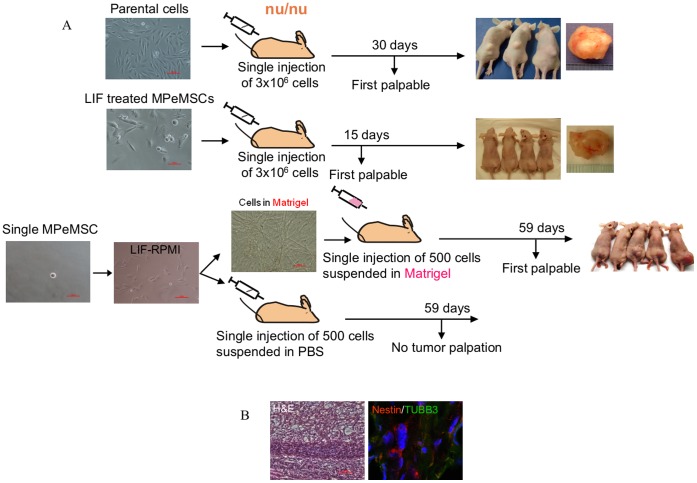
Malignant peritoneal mesothelioma stem cells (MPeMSCs) and endothelial lineage-induced cells promote early tumor growth. (**A**) Parental cells from which MPeMSCs derived were injected subcutaneously (n = 8) into the right flank of athymic nude mice (nu/nu). First palpable tumor appeared on day 30 after tumor cell injection. Similar injections of MPeMSCs (n = 8) generated palpable tumors on day 15 (end-stage tumors are shown). In limiting dilution experiments, cells derived from a single MPeMSC were divided and grown either in a Matrigel plate to induce endothelial differentiation or in a regular cell culture plate with LIF containing medium. Five hundred cells dissociated from the first group were resuspended in Matrigel and injected subcutaneously (n = 8). Tumors were first found by palpation on day 59 (mice with 60 days of tumor growth after tumor palpation are shown) whereas similar injections of MPeMSCs (n = 10) resuspended in PBS did not generate tumors. (**B**) Hematoxylin and eosin (H&E) staining and immunofluorescence of tumor tissues. Early tumors generated from endothelial lineage-induced cells express neuronal markers; NES and TUBB3.

Our initial studies using MPeM parental cells have shown that injections of 3×10^6^ Meso-CL1 cells were able to generate both subcutaneous and intraperitoneal tumors in immunocompromised mice whereas Meso-CL2 and Meso-CL3 were non-tumorigenic. In the subcutaneously injected mice, Meso-CL1 cells consistently generated first palpable tumor on day 30 after tumor cell transplantation whereas similar injections of MPeMSCs took only 15 days to develop the first palpable tumor. Conversely, transplantation of neural lineage-induced cells showed tumor palpation on day 35 demonstrating that both parental cells and neural differentiated cells had similar tumorigenic potential. Studies have shown that injections of tumor cells suspended in Matrigel have higher frequencies of tumor formation [22]. Our *in vitro* studies demonstrated that Matrigel promoted endothelial differentiation and activation of angiogenic factors in endothelial differentiated cells. Therefore, it is possible that a small number of MPeMSCs could generate tumors within an endothelial niche. To test the significance of endothelial cell differentiation of MPeMSCs on tumor growth, we performed limiting dilution experiments. Five hundred cells derived from a single unselected MPeMSC injected with Matrigel generated palpable tumors in mice on day 59 after tumor cell transplantation whereas mice received an equal number of cells suspended in PBS did not grow tumors during a two month time period (**Fig. 5A**) indicating that endothelial differentiation of CSCs is central to tumor growth. To test *in vivo* transdifferentiation of endothelial cells derived from MPeMSCs, early tumors derived from endothelial lineage-directed cells co-injected with Matrigel were analyzed for the expression of NES and TUBB3 and found that cells express both neuronal proteins (**Fig. 5B**).

## Discussion

The important finding from this study is the identification of CSCs with characteristic asymmetric stem cell division and tumorigenicity in human MPeM tumors. To date CSC markers have been widely used to identify and isolate stem cells from tumors. However, no stem cell marker has been proven to be a universal marker for CSCs. Defined culture of MPeMSCs derived from patient tumors promoted self-renewal, colony formation and multilineage differentiation confirming a stem cell phenotype. The distinctive feature of MPeMSCs over other CSCs is that cells rarely form clonal spheres in regular adherent cell culture and therefore the individual cell division pattern can be visualized using microscopy, which helps to identify and isolate CSCs. Asymmetric cell division is characteristic of embryonic stem cells and our time-lapse studies showed that MPeMSCs divide asymmetrically and the daughter cells inherit distinct cytoplasmic components [23] to form stem cells and differentiating cells. This observation was further supported by pulse-chase BrdU labeling procedure and found that the template DNA retains within the daughter stem cell. Moreover, we found symmetric cell division in MPeMSCs generating cells with approximately equal sizes. Apart from these characteristic stem cell divisions, MPeMSCs show a different mechanism for stem cell proliferation by generating cells encapsulated within a membranous outer covering. These cells generally float in culture, and display both asymmetric and symmetric division. The significance of capsular stem cell formation needs to be further investigated. It is possible that the formation of such encapsulated cells is niche homeostasis dependent and that allows CSCs to proliferate rapidly and survive in adverse niche conditions. The unique morphologic characteristics of these cells can also be utilized to identify CSCs *in vitro*. The derived MPeMSCs show significant expression of MSLN, a prominent marker for mesothelioma [24] indicating that CSCs originated from the parental mesothelioma cells. The stem cell markers tested in both parental cells and MPeMSCs show similarities in their expression, except for the MYC oncogene, and this consistency in gene expression indicates that parental cells also have potential for dedifferentiation [5]. The MYC oncoprotein which is activated in several tumor types [25] is significantly upregulated in MPeMSCs; MYC gene is important for stem cell proliferation and for the maintenance of pluripotent properties of stem cells [26], [27].

Recent reports on stem-like cells derived from glioblastoma suggest that CSCs differentiate into endothelial cells during tumor growth [28], [29]. To further confirm the presence of stem cell phenotype in MPeM, we tested multipotency of MPeMSCs by growing them in multiple differentiation conditions. Cells were first cultured in serum-minimized neuronal differentiation conditions and found that MPeMSCs differentiated into cells with typical neural morphology and expressed neuronal markers. We identified NES as an important neuronal stem cell marker in MPeMSCs and its expression was also found in xenograft tumors indicating that neuronal differentiation of endothelial cells is possible during tumor growth. Similarly, the endothelial stem cell marker VEGFR2 expression was also markedly high in MPeMSCs; in a matrix niche cells differentiated into endothelial cells [9] with typical tubular networks suggesting MPeMSCs are intrinsically programmed for endothelial differentiation. This direct contribution of MPeMSCs to the tumor vasculature may be important for the rapid and diffuse tumor growth found in MPeM patients. Neural stem cells are ectodermal in origin whereas endothelial cells originate from the mesoderm. However, endothelial-like cells can be derived from neural stem cells [30]. In our studies (unpublished data) when neural differentiated cells were seeded in a Matrigel plate, cells differentiated into endothelial cells with typical tubular networks indicating transdifferentiation of lineage-committed cells. It has been reported that endothelial cells transdifferentiate into mesenchymal cells to induce stem-like properties [31]. Clinical studies have shown that benefits from anti-angiogenic therapy for cancer are limited and in many cases anti-angiogenic therapies result in an initial response followed by tumor relapse [32]. In this context vascular differentiation of CSCs residing within the tumors may have a central role in rapidly replenishing endothelial cells.

No study so far has addressed that a particular cell type initiates tumor growth in patients. Our initial xenotransplantation studies using leukemia inhibitory factor (LIF) treated undifferentiated MPeMSCs showed an early tumor induction in mice proving that MPeMSCs are tumorigenic. Although MPeMSCs do not form colony spheres in adherent cell culture, single cell culture in a matrix plate generated colony spheres showing the stem cell phenotype of the derived cells. In this study, we generated a CSC tumor model using a limited number of cells proliferated from a single MPeMSC. Data from this study, when viewed together with *in vitro* evidences support a model where MPeMSCs resuspended in Matrigel promote aggressive tumor growth principally due to the endothelial differentiation of CSCs because all injected mice developed palpable tumors approximately 60 days of tumor cell injection. Conversely, tumor palpation was not evident in mice injected with undifferentiated MPeMSCs resuspended in PBS. Although cells with tumorigenic potential are equally distributed in two groups, the matrix components promoted colony formation and the rapid induction of endothelial lineage. In tumors endothelial cells dedifferentiate or transdifferentiate to form more stem-like cells or other distinct cell types that may contribute to an early tumor growth. It has been shown that the microenvironment surrounding the neovasculature of tumors is highly proliferative and CSCs have been found in close proximity to endothelial cells [33]. Together our data suggest that multipotency and plasticity of MPeMSCs may be a contributing factor for MPeM tumor growth in patients. Further examination of factors associated with CSC proliferation and lineage differentiation may provide important insights into the development of MPeM and may provide a foundation for the development of more effective therapeutic interventions.

## Materials and Methods

### Cell Lines, Cell Culture and Isolation of MPeMSCs

MPeM cell lines (Meso-CL1, Meso-CL2 and Meso-CL3) were derived from human MPeM tumors collected on institution review board approved protocols of the National Cancer Institute and informed consent was obtained from all patients for the use of tumor samples, including the generation of cell lines [15]. Cells were cultured in RPMI-1640 (RPMI; Gibco) supplemented with 10% v/v FBS (Atlanta Biologicals) under standard cell culture conditions in a humidified 5% CO2 incubator at 37°C. Cells were confirmed as mesothelioma by immunohistochemistry for known mesothelioma markers and by electron microscopy and were characterized for *in vitro* growth characteristics and *in vivo* xenograft growth. Although all three MPeM cell lines generated floating stem cells, Meso-CL1, the PTEN ^(neg)^ cell line with potential to generate both subcutaneous and intraperitoneal tumors in athymic nude (nu/nu) mice was used for the study. We activated stem cell generation in MPeM cells by seeding them at sub-confluent density to minimize cell-to-cell anchorage in 5% v/v FBS-RPMI (serum-reduced medium). Under these conditions the MPeMSCs present in the parental cell population generate floating stem cells which were then collected and cultured in the presence of LIF to maintain them in an undifferentiated state. The floating MPeMSCs collected from the LIF treated cell culture was used for subsequent experiments. For all experiments MPeMSCs were cultured in the absence of LIF unless otherwise indicated.

### Short-Time Live Cell Imaging and Immunofluorescence

To determine that the isolated floating cells were CSCs, we first studied stem cell division in living cells by time-lapse studies using microscopy (Olympus IX71); pictures were captured using CellSens software. For immunofluorescence, cells were fixed with 4% paraformaldehyde for 10 min at room temperature, and then treated with 0.2% Triton X-100 (Sigma) in PBS for 10 min except for the detection of cell surface proteins. Cells were washed, with PBS and blocked with 5% BSA in PBS containing 0.25% NP-40 for 30 min at room temperature. Cells were then incubated with any of the following primary antibodies: Alexa Fluor conjugated monoclonal anti-α-tubulin (1∶1000; Millipore), Alexa Fluor conjugated Phalloidin (1∶1000; Invitrogen), Hoechst 33342 (1∶5000; Sigma), anti-nestin (anti-NES, 1∶250; Millipore), anti-βIII-tubulin (anti-TUBB3; 1∶1000; Covance), anti-vascular endothelial growth factor receptor (anti-VEGFR) 2 (1∶200; Cell Signaling), anti-CD133/1-allophycocyanin (APC, 1∶100; Miltenyi Biotech). The following secondary antibodies from Molecular Probes (Invitrogen) were used: Alexa Fluor conjugated mouse or rabbit anti-IgG (1∶1000). Secondary antibodies alone or mouse IgG1 served as control for unspecific binding. Images were captured using either Olympus CKX41 fluorescent microscope equipped with F-View II CCD digital camera or using Olympus 1×81 microscope with a Fluoview-1000 confocal laser scanner.

### BrdU Labeling and Chase

To further determine the asymmetric cell division in MPeMSCs, we sought to test the segregation of the template DNA during mitotic cell division using 5-bromo-2-deoxyuridine (BrdU) pulse-chase [34]. Briefly, MPeMSCs were treated with 1 µM BrdU (Sigma) for several passages to ensure that DNA strands were labeled with BrdU in vast majority of cells. After a long pulse BrdU were removed from the cell culture and were examined for the BrdU labeling pattern during cell division. Cells were fixed in 70% ethanol and incubated in 2N HCl containing 0.5% Triton X-100 for 1 h. and blocked with 5% BSA containing 0.25% NP-40. Cells were washed and incubated with anti-BrdU-FITC antibody (BD Biosciences) overnight at 4°C, washed and counterstained with DAPI. BrdU labeling pattern in cells were viewed using Olympus 1×81 microscope with a Fluoview-1000 confocal laser scanner.

### RNA Preparation, Real-time Quantitative PCR and Western Blotting

Total RNA was extracted from cells using Trizol (Invitrogen) and pre-treated with DNase. A total of 1 µg RNA was reverse transcribed into cDNA with Reverse Transcriptase III (Invitrogen) according to the manufacturer’s instructions. Quantitative real-time PCR (qPCR) was performed with 7500 real-time PCR system using buffer master mix and pre-made primer and probe mix (Applied Biosystems) for phosphatase and tensin homolog (PTEN), mesothelin (MSLN), homeobox protein NANOG (NANOG), sex determining region Y-box-2 (SOX-2), octamer-binding transcription factor 4 (Oct-4), myelocytomatosis oncogene (c-MYC), Kruppel-like factor 4 (KLF4), NES, TUBB3, brain expressed X-linked 1 (BEX1), vascular endothelial growth factor (VEGF) A (VEGFA), VEGFC, VEGFR2, VEGFR3, von Willebrand factor (vWF), interleukin-8 (IL-8) and fatty acid binding protein 4 (FABP4). Peroxisome proliferator activated receptor-γ (PPARγ) qPCR was performed using SYBR premix (Applied Biosystems) and the following primer set; forward primer 5′ ACA CAA TGC TGG CCT CCT TG- 3′, reverse primer 5′AAA AGG CTT TCG CAG GCT CT 3′. β-actin was used for normalization of the data. Western blotting was performed using anti-PTEN antibody (Cell Signaling) and anti-β-actin antibody (Sigma). HRP-labeled anti-rabbit IgG (Cell Signaling) or anti-mouse IgG (GE Healthcare) was used as secondary antibodies.

### Flow Cytometry

MPeMSCs were dissociated using Trypsin, washed with PBS and incubated in FcR blocking reagent (Miltenyi Biotech). Cells were then incubated with any of the following primary antibodies specific for CD133/2-APC (Miltenyi Biotech), CD31-phycoerythrin (PE, BD Biosciences), CD144-PE (R&D Systems) or corresponding isotype control antibodies as per the manufacturers’ protocols. Flow cytometry was performed at our flow cytometry core facility using FACS Canto II (BD Biosciences).

### Neuronal, Endothelial and Adipose Lineage Induction in MPeMSCs

For neuronal differentiation, MPeMSCs were cultured in serum-minimized medium (1% v/v FBS-RPMI) containing 10 µM trans-retinoic acid (Sigma-Aldrich) and were cultured in the medium for 3 days followed by the addition of neural differentiation medium containing serum-reduced Dulbecco’s Modified Eagle’s Medium (DMEM), neurobasal medium (3∶1), N2 and B-27 supplements (Invitrogen), FGF-2 (5 ng/ml, Cell Signaling) and FGF8b (50 ng/ml, R&D systems) and were maintained in the medium for 8 days. Cells were fed every third day, the neural differentiation was analyzed microscopically and by the expression of neuronal markers using immunofluorescence and qPCR. Cells were then maintained in the neural differentiation medium without FGF [35] and the neurospheres generated from neural differentiated cells were collected and cultured for several generations in serum-minimized neural differentiation medium.

For endothelial differentiation, MPeMSCs were cultured on a Matrigel (BD Biosciences) coated cell culture plate in the presence of serum-reduced medium. To demonstrate colony sphere formation, cells were deposited in single cell per well in a 24-well Matrigel coated plate containing serum-reduced RPMI and expanded for 10 days. Endothelial tubular networks were pictured using Nikon TS100. Expression of endothelial progenitor markers and mature endothelial cell specific markers were assessed using immunofluorescence microscopy and qPCR.

For the adipose cell differentiation MPeMSCs were grown in serum-reduced RPMI:DMEM (1∶1) medium containing 0.5 µM trans-retinoic acid (Sigma) and 50 nM insulin (Sigma) for 2 days followed by the addition of adipose differentiation medium containing 170 nM insulin, 2 nM triiodothyronine and 0.5 µM rosiglitazone [36]. Cells were maintained in the medium for 5 days and neutral lipid accumulation was detected in 4% formaldehyde fixed cells using Oil red O (Sigma) staining. Control cells were maintained in the RPMI-DMEM medium. Lipid staining was observed using Olympus IX71 microscope. Adipose specific gene expression was determined using qPCR in both control and adipose differentiated cells.

### Dil-AcLDL Assay

Endothelial lineage-induced cells were treated with 10 mg ml^−1^ Dil-labeled acetylated low-density lipoproteins (AcLDL, Molecular Probes) and grown under standard cell culture conditions for 4 h. Cells were analyzed using an Olympus CKX41 fluorescent microscope equipped with F-View II CCD digital camera.

### Mouse Models

Parental cells from which MPeMSCs derived were cultured in 10% v/v FBS containing RPMI whereas all other groups of cells were cultured in serum-reduced medium unless specified otherwise. MPeMSCs were cultured in LIF containing medium (3 ng/ml) to inhibit CSC differentiation and then briefly cultured in serum-reduced medium without LIF before cell preparation for injection. Neural lineage-induced cells were cultured in neural differentiation medium. Cells were dissociated with trypsin, washed, counted, resuspended in PBS and 3×10^6^ cells per mouse were injected by 25-gauge needle subcutaneously into the right flank of female athymic nude mice of 4–5 weeks old. For the limiting dilution experiments, MPeMSCs were deposited in single cell per well in a 24-well cell culture plate containing serum-reduced RPMI and expanded in the presence of LIF. Wells were visually confirmed to contain single cell. On day 5 cells were dissociated with trypsin and one portion of cells was seeded in a Matrigel plate to induce endothelial differentiation and the other portion was cultured in a regular cell culture plate. Cells were fed with either serum-reduced RPMI or RPMI-LIF respectively, every 3^rd^ day, cells were dissociated with trypsin, counted, and 500 cells from the respective group were re-suspended in fresh Matrigel or in PBS and injected per mouse subcutaneously. Tumor formation was evaluated regularly by tumor palpation (tumor volume∼20 mm^3^) and tumor diameters were measured with calipers to determine progressive tumor growth. Tumors were surgically removed when a few mice from each group showed signs of distress. Early tumors (20 days after tumor palpation) were collected from endothelial-lineage induced cells injected mice for immunofluorescence studies.

### Ethics Statement

Animals were housed and cared for in accordance with the guidelines for animal welfare and all animal experiments were performed in accordance with protocols approved by the University of Maryland Institutional Animal Care and Use Committee (IACUC).

### Tissue Histopathology and Immunofluorescence

Histopathology and immunostaining was performed on fresh frozen tumor tissues. Frozen tumors were embedded in Optimal Cutting Temperature compound (O.C.T, Sakura Finetek) and sectioned on a freezing cryostat (Leica). Tissue sections were bathed in 1% BSA containing PBS and fixed in 4% paraformaldehyde for immunostaining. Slides were rinsed in PBS and blocked with 5% BSA and 0.5% NP-40 in PBS for 1 h to reduce non-specific antibody binding. Incubation with anti-NES antibody (1∶200; Millipore) and anti-TUBB3 antibody (1∶1000; Covance) was performed overnight at 4°C followed by fluorescent-labeled secondary antibodies for 1 h at room temperature. Secondary antibodies alone or mouse IgG1 served as control for unspecific binding. Slides were counter stained with Hoechst stain for 10 min at room temperature and mounted in fluorescent mounting solution (DAKO). Images were acquired using Olympus 1×81 microscope with a Fluoview-1000 confocal laser scanner.

### Statistical Analysis

The Student t test was used to assess difference between the groups in qPCR studies. Results are expressed as mean ± SEM.

## Supporting Information

Figure S1 **PTEN expression in malignant peritoneal mesothelioma (MPeM) cell lines (CLs).** **(A)** Relative mRNA expression of PTEN was measured by real time qPCR and **(B)** Immunoblotting of whole cell extract using PTEN specific antibody. β-actin was used as control. Meso-CL1 is PTEN negative whereas Meso-CL2 and Meso-CL3 are PTEN positive. Bar diagrams are expressed as mean ±SEM.
(TIF)
