# Supplementary figures and images for: Multipotent Cancer Stem Cells Derived from Human Malignant Peritoneal Mesothelioma Promote Tumorigenesis
Source: PLoS One. 2012 Dec 28;7(12):e52825. doi: 10.1371/journal.pone.0052825 (PMC3532216; doi:10.1371/journal.pone.0052825)

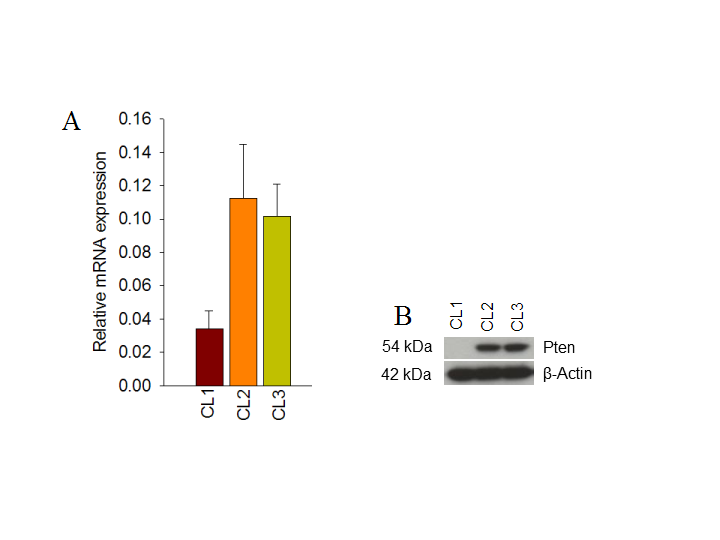

Supplement: Figure S1 — PTEN expression in malignant peritoneal mesothelioma (MPeM) cell lines (CLs). (A) Relative mRNA expression of PTEN was measured by real time qPCR and (B) Immunoblotting of whole cell extract using PTEN specific antibody. β-actin was used as control. Meso-CL1 is PTEN negative whereas Meso-CL2 and Meso-CL3 are PTEN positive. Bar diagrams are expressed as mean ±SEM. (TIF) [file pone.0052825.s001.tif]
